# Establishment and solution of a three-zone radial composite well test model for mixed gas drive production wells

**DOI:** 10.1038/s41598-025-00645-8

**Published:** 2025-05-07

**Authors:** Xiaoliang Zhao, Yu Cao

**Affiliations:** 1 Department of Petroleum Engineering, China University of Petroleum (Beijing), Beijing, 102200 China; 2 State Key Laboratory of Petroleum Resources and Engineering, China University of Petroleum (Beijing), Beijing, 102200 China; 3 Department of Petroleum and Natural Gas Engineering, LiaoNing Petrochemical University, Fushun, 113001 China

**Keywords:** Mixed gas drive, Dynamic inversion, Nonlinear seepage, Well test analysis, Parameter sensitivity analysis, Crude oil, Fluid dynamics

## Abstract

In the process of gas flooding, the underground miscible law and oil displacement characteristics are complex, and there is no effective evaluation method. As an effective method to invert reservoir parameters and analyze seepage law, well test can be used to analyze the law of underground gas and oil action. This study focuses on the mechanism of gas-crude oil interaction, and establishes a three-zone radial composite well test model including interface skin effect and power-law change of physical properties of transition zone. The model innovatively introduces the interface coefficient to characterize the phase transition effect of the regional boundary, which is solved by dimensionless transformation, Laplace transformation and Stehfest numerical inversion method. Get the fluid phase distribution evaluation chart in the process of gas flooding. The results show that the radius of the crude oil zone (R_1_) determines the duration of the radial flow, the radius of the gas-oil transition zone (R_2_) regulates the seepage range, the power law index (θ) controls the derivative curve shape, and the energy storage index (I) has a weak influence on the curve shape. This study establishes a theoretical framework for dynamic monitoring of miscible gas drive wells and nonlinear reservoir parameter inversion.

## Introduction

The study of laboratory conditions shows that gas flooding has the mechanism of reducing interfacial tension and improving the physical properties of crude oil, so as to achieve the purpose of improving reservoir recovery. However, in the process of on-site gas injection, whether the underground flow characteristics of the injected gas match with the indoor research needs to be verified. Well test is the key method to monitor the underground flow. However, the mathematical description of complex fluid dynamic distribution is a difficult problem in gas drive reservoir. The traditional well test model is difficult to accurately describe the nonlinear flow characteristics^1^.

The method of determining the physical parameters of the wellbore, reservoir, and fluid according to the pressure data of real test wells is collectively referred to as the pressure dynamic inversion method, which is also called unstable pressure analysis ( PTA )^2^ This method combines the dynamic characteristic analysis method of bottom hole pressure and the typical well test curve fitting method. The analysis object is the well test pressure data of oil and gas wells. At present, there are many international studies on the dynamic inversion method of pressure. Horner, Miller et al.^3^ have proposed the ‘Horner method ‘and ‘semi-logarithmic curve method ‘, which have been used until now, and then gradually become one of the important analysis methods of pressure dynamic inversion. This kind of method mainly determines the physical parameters of reservoir and fluid by analyzing the relationship between pressure logarithm value and time logarithm value. Ramey et al.^4^, based on the ' semi-logarithmic curve method ' and ' Horner method ‘, have obtained well test curve interpretation charts for different reservoirs. This chart has made outstanding contributions to the popularization and application of the above two methods, and opened a new chapter in well test interpretation technology. Gringarten et al.^5^ further analyzed the relationship between the dimensionless pressure parameter group and the dimensionless time parameter group, and obtained the pressure curve chart of single / double pore media and fracturing wells, which greatly improved the efficiency of pressure curve fitting. In addition, Gringarten et al.^5^ laid a solid foundation for the ' modern well test interpretation method ‘, making the pressure dynamic inversion method enter a new stage of modern well test interpretation. Based on the previous research results, Bourdet et al.^6^ further proposed a new method of pressure derivative curve well test interpretation, which can more accurately identify the reservoir fluid flow stage than the pressure curve well test interpretation method. Since Bourdet et al.improved the modern well test dynamic inversion technology, the parameter adjustment fitting dynamic inversion method has always run through the whole well test interpretation. Based on the previous research, Jia et al.^7^ proposed a multi-objective constrained fracture parameter inversion method based on microseismic data and fracturing construction data. By introducing the crack tip expansion domain and GSA algorithm to optimize the fracture expansion direction, combined with the number of constrained nodes of fracturing construction parameters, a data-driven dynamic inversion model is constructed. The fitting rate of the 18-stage fracturing model of the whole well is 90%, and the calculation takes only 5 minutes. This method breaks through the limitations of traditional mechanical models and provides an efficient solution for the dynamic description of fracture morphology in unconventional oil and gas reservoirs. The study of Huang et al.^8^ pointed out that for the inversion method of fracture network parameters in shale gas reservoirs, the existing technology mainly depends on laboratory experiments, microseismic data inversion and production data history matching. Although the laboratory method can restore the physical properties of rock, the simulation size is different from the actual one. Microseismic inversion describes fracture morphology by fractal theory, but does not fully consider the influence of reservoir stress and natural fracture heterogeneity. Production data fitting combines analysis model and numerical simulation, but it is mostly based on simplified fracture hypothesis, which is difficult to describe complex networks.

A three-zone radial composite well test model for the fluid distribution characteristics of production wells in the later stage of gas flooding. Fevang and Whitson pioneered the establishment of a three-zone flow mathematical model for reservoir description, revealing the inhibition of condensate saturation gradient change on gas relative permeability near wellbore^7^. On this basis, Xu and Lee proposed a radial three zone composite two phase flow model. The pseudo pressure function was used to quantify the influence of multiphase flow effect on pressure transient curve, which provided a theoretical basis for well test interpretation^8^. However, Bozorgzadeh and Kgogo found that the model is only applicable to light and moderately enriched condensate gas reservoirs, while gas-rich reservoirs only show two-zone characteristics, revealing the applicability boundary of the model^9,10^. In view of the complex flow characteristics of horizontal wells, Yin et al. innovatively combined the three-zone model with the Warren-Root dual-porosity model, introduced the stress sensitivity parameter of fracture permeability, and constructed a semi-analytical model of horizontal wells. The analytical accuracy of the model for the reverse condensation effect was verified by CMG numerical simulation, which made up for the shortcomings of the traditional model in the dynamic characterization of the horizontal well near the well area^11^. In terms of experimental research, Liang et al. confirmed that reservoir connectivity is the key control factor affecting the application effect of the three-zone model through the analysis of water flooding response characteristics. The production characteristics of ' four increase and one decrease ' proposed by Liang et al. provide a dynamic verification basis for model parameter inversion^12^. It is worth noting that LaFitte compared the GOR variation of gas flooding and water flooding, and found that although gas flooding significantly increases the production well rate, it is easy to cause gas channeling. This conclusion echoes the formation mechanism of the expansion rate in the third zone ( single-phase flow zone in the far well zone ) predicted by Xu model^13^. Wang et al. ' s latest research quantitatively revealed that near gas flooding can achieve stable production when BHP > 30 MPa by systematic experiments of adjusting production bottom hole flowing pressure ( BHP ), which provides an operating threshold for the engineering control of the transition zone radius in the three-zone model^14^. However, there are still gaps in the systematic study of the three-zone composite model: on the one hand, the dynamic changes of the physical parameters in the transition zone lack quantitative analysis; on the other hand, parameter sensitivity research is insufficient. The traditional model assumes that the physical properties of each zone are constant, which is difficult to reflect the nonlinear seepage characteristics of the transition zone in actual mining, resulting in deviations in the sensitivity evaluation of key parameters such as crossflow coefficient and permeability ratio between inner and outer zones.

To address the limitations of traditional models in capturing nonlinear seepage and dynamic parameter variations in transition zones, this study develops a three-zone radial composite well test model. The model incorporates: (1) power-law relationships to characterize nonlinear changes in fluid mobility and storativity in the transition zone; (2) interfacial skin coefficients to account for pressure jump effects at zone boundaries. By solving the model using Laplace transform and Stehfest inversion, we aim to provide a theoretical framework for dynamic monitoring of miscible gas flooding processes and improve the accuracy of parameter inversion in heterogeneous reservoirs.

## Radial composite seepage physical model of production well

**Fig. 1:**
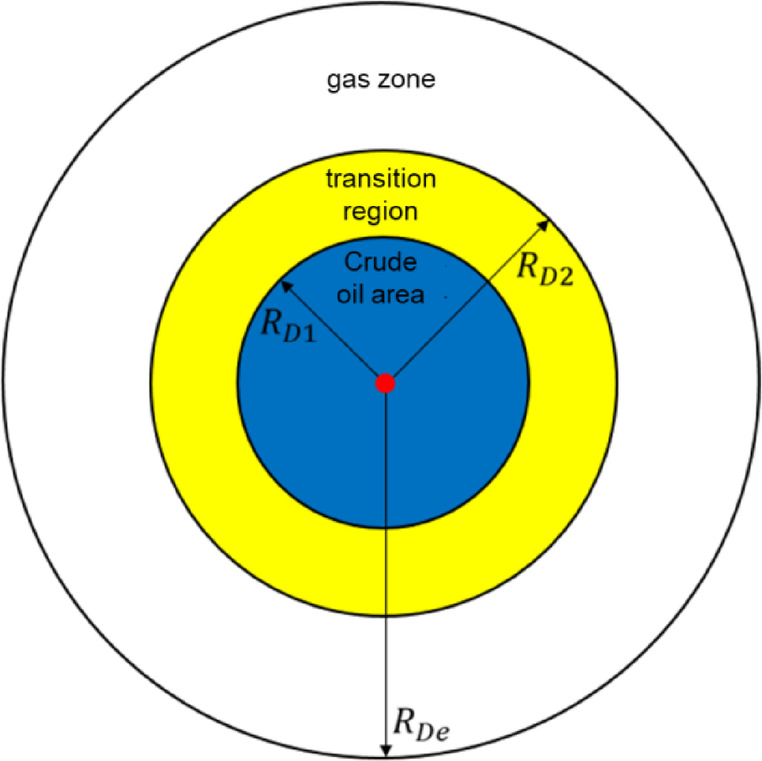
Physical model of late production well with triple-zone composite fluid.

**Fig. 2:**
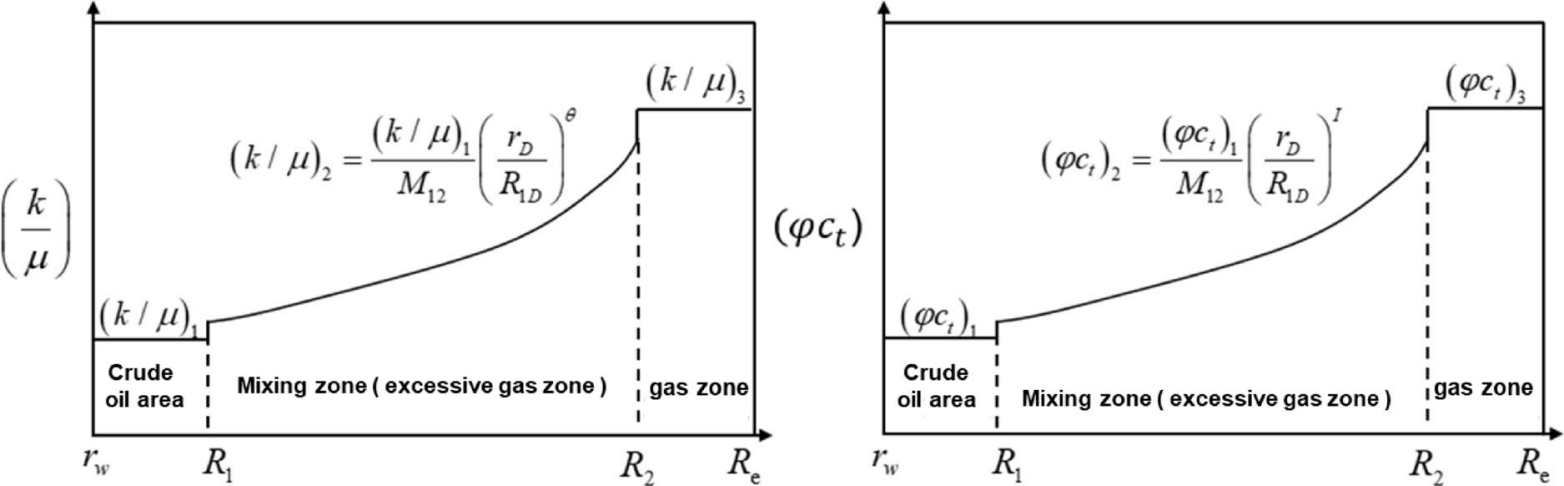
Mobility and storativity coefficient distribution of late production well.

Corresponding to the fluid distribution characteristics of the injection wells in the later stage of gas flooding, the inner zone of the production well is the reservoir crude oil (referred to as the crude oil zone), the middle zone is the mixed oil and gas in the transition zone (referred to as the transition zone), and the outer zone is the pure injection gas (referred to as the pure gas zone)^15^. Therefore, according to the characteristics of fluid distribution and physical property change in the late stage of gas flooding in production wells, a three-zone radial composite well test model for production wells in the late stage of mixed gas flooding can be established, as shown in Fig. 1. The assumptions of the physical model in the later stage of mixed gas flooding in production wells are as follows:

1. Consider the influence of wellbore reservoir effect and skin effect in production wells^16,17^;
2. In the later stage of mixed gas flooding, the displacement efficiency of the near-injection well zone is extremely high, which makes the crude oil in the crude oil area, the mixed oil and gas in the transition zone, and the pure gas zone in the production well as the center of the circle expands outwards in turn^18,19^. According to the distribution characteristics of fluid physical properties, there is an exponential relationship between the fluidity/storage capacity coefficient of mixed oil and gas and the radial distance in the transition zone, as shown in Fig. 2, Eq. (1), and Eq. (2)^20,21^.Among them, $\:{\uptheta\:}$ determines the change trend of fluid flow in the transition zone with radial distance, and I describes the change trend of energy storage coefficient in the transition zone with radial distance. Both of them are the core parameters to characterize the nonlinear changes of fluid physical properties in the transition zone.
3. According to the pressure characteristics at different interfaces: the pressure at the interface decreases sharply due to the drastic changes in the fluid composition at the interface of each zone. The “interfacial skin” coefficient was used to describe the additional pressure drop characteristics at the interface of each zone (Fig. 2).
4. The fluids in each zone of the formation are micro-compressible, ignoring the influence of gravity and capillary forces.
5. The temperature is constant during the seepage process, the fluid follows the Darcy seepage principle, and the stratum is infinitely large;
6. The reservoir is horizontal, homogeneous, and of equal thickness, and the initial pressure is equal everywhere.

1 $$\:{\left(k/\mu\:\right)}_{2}=\frac{{\left(k/\mu\:\right)}_{1}}{{M}_{12}}{\left(\frac{{r}_{D}}{{R}_{1D}}\right)}^{\theta\:}$$

2 $$\:{\left(\phi\:{c}_{t}\right)}_{2}=\frac{{\left(\phi\:{c}_{t}\right)}_{1}}{{F}_{12}}{\left(\frac{{r}_{D}}{{R}_{1D}}\right)}^{I}$$

Where:

$\:(k/\mu\:{)}_{2}$—Fluid fluidity in the transition zone in the late stage of gas flooding in production wells, $\:\text{mD}\cdot\:\text{c}{\text{p}}^{\text{-1}}$;

$\:(k/\mu\:{)}_{1}$—Fluid fluidity in the crude oil zone in the late stage of gas flooding in production wells, $\:\text{mD}\cdot\:\text{c}{\text{p}}^{\text{-1}}$;

$\:\theta\:$—Power law variation index of fluid flow in the transition zone in the late stage of gas flooding in production wells, dimensionless;

$\:{M}_{12}$—Interface fluidity ratio between crude oil zone and transition zone in the later stage of gas flooding in production wells, dimensionless;

$\:(\phi\:{c}_{t}{)}_{2}$—Fluid storage capacity coefficient in the transition zone in the later stage of gas flooding in production wells, $\:\text{MP}{\text{a}}^{\text{-1}}$;

$\:(\phi\:{c}_{t}{)}_{1}$—Crude oil storage capacity coefficient in the late stage of gas flooding of production wells, $\:\text{MP}{\text{a}}^{\text{-1}}$;

$\:I$—Power law variation index of storage capacity coefficient in the transition zone in the late stage of gas flooding in production wells, dimensionless;

$\:{F}_{12}$—Storage-capacity ratio at the interface between the crude oil zone and the transition zone in the late stage of gas flooding in production wells, dimensionless;

$\:{r}_{D}$—dimensionless distance from the reservoir to the injection well, dimensionless;

$\:{R}_{1D}$—Dimensionless radius of crude oil area, dimensionless.

## Mathematical model of radial unstable seepage in production wells

### Establishment of mathematical models

#### Seepage differential equations

3 $$\:\text{C}\text{r}\text{u}\text{d}\text{e}\:\text{o}\text{i}\text{l}\:\text{z}\text{o}\text{n}\text{e}: \, \frac{1}{r}\frac{\partial\:}{\partial\:r}\left({\left(\frac{k}{\mu\:}\right)}_{1}r\frac{\partial\:{p}_{1}}{\partial\:r}\right)=\frac{{\left(\varphi\:{c}_{t}\right)}_{1}}{3.6}\frac{\partial\:{p}_{1}}{\partial\:t},\begin{array}{cc}&\:{r}_{w}\le\:r<{R}_{1}\end{array}$$

4 $$\:\text{T}\text{r}\text{a}\text{n}\text{s}\text{i}\text{t}\text{i}\text{o}\text{n}\:\text{Z}\text{o}\text{n}\text{e}: \, \frac{1}{r}\frac{\partial\:}{\partial\:r}\left({\left(\frac{k}{\mu\:}\right)}_{2}r\frac{\partial\:{p}_{2}}{\partial\:r}\right)=\frac{{\left(\varphi\:{c}_{t}\right)}_{2}}{3.6}\frac{\partial\:{p}_{2}}{\partial\:t},\begin{array}{cc}&\:{R}_{1}\le\:r<{R}_{2}\end{array}$$

5 $$\:\text{P}\text{u}\text{r}\text{e}\:\text{g}\text{a}\text{s}\:\text{z}\text{o}\text{n}\text{e}: \, \:\frac{1}{r}\frac{\partial\:}{\partial\:r}\left({\left(\frac{k}{\mu\:}\right)}_{3}r\frac{\partial\:{p}_{3}}{\partial\:r}\right)=\frac{{\left(\varphi\:{c}_{t}\right)}_{3}}{3.6}\frac{\partial\:{p}_{3}}{\partial\:t},\begin{array}{cc}&\:{R}_{2}\le\:r<{R}_{e}\end{array}$$

#### Inner boundary conditions

6 $$\:\underset{r\to\:{r}_{w}}{lim}\left(r\frac{\partial\:{p}_{1}}{\partial\:r}\right)=\frac{1.842\times\:1{0}^{-3}{\mu\:}_{o}{B}_{o}q}{kh}$$

#### Outer boundary conditions

7 $$\:\text{I}\text{n}\text{f}\text{i}\text{n}\text{i}\text{t}\text{e}\:\text{s}\text{t}\text{r}\text{a}\text{t}\text{a}: \, \:{p}_{3}(r\to\:\infty\:,t)={p}_{i}$$

8 $$\:\begin{array}{c}Circular\:constant\:pressure\:boundary: \, {p}_{3}\left(r={R}_{e},t\right)={p}_{i}\end{array}$$

9 $$\:\text{C}\text{i}\text{r}\text{c}\text{u}\text{l}\text{a}\text{r}\:\text{C}\text{l}\text{o}\text{s}\text{e}\text{d}\:\text{B}\text{o}\text{u}\text{n}\text{d}\text{a}\text{r}\text{y}:\:\frac{\partial\:{p}_{3}(r={R}_{e},t)}{\partial\:r}=0$$

#### Interface continuity condition

The continuous conditions of the mixed oil and gas interface between the crude oil zone and the transition zone follow the flow equality criterion, and the pressure change characteristics of the interface between the crude oil zone and the transition zone are described by the “interfacial skin” coefficient^22^.

10 $$\:\left\{\begin{array}{c}{p}_{1}(r={R}_{1},t)={p}_{2}(r={R}_{1},t)-{\left({S}_{1}r\frac{\partial\:\left({p}_{2}\right)}{\partial\:r}\right)}_{r={R}_{1}}\\\:{\left(\frac{k}{\mu\:}\right)}_{1}\frac{\partial\:\left({p}_{1}\right)}{\partial\:r}(r={R}_{1},t)={\left(\frac{k}{\mu\:}\right)}_{2}\frac{\partial\:\left({p}_{2}\right)}{\partial\:r}(r={R}_{1},t)\end{array}\right.$$

The continuous conditions at the interface between the mixed oil and gas zone and the pure gas zone in the transition zone follow the flow rate equality criterion, and the pressure change characteristics at the interface between the transition zone and the pure gas zone are described by the “interfacial skin” coefficient.

11 $$\:\left\{\begin{array}{c}{p}_{2}(r={R}_{2},t)={p}_{3}(r={R}_{2},t)-{\left({S}_{2}r\frac{\partial\:\left({p}_{3}\right)}{\partial\:r}\right)}_{r={R}_{2}}\\\:{\left(\frac{k}{\mu\:}\right)}_{2}\frac{\partial\:\left({p}_{2}\right)}{\partial\:r}(r={R}_{2},t)={\left(\frac{k}{\mu\:}\right)}_{3}\frac{\partial\:\left({p}_{3}\right)}{\partial\:r}(r={R}_{2},t)\end{array}\right.$$

#### Initial

The pressures are equal everywhere in the reservoir at the initial moment:

12 $$\:{p}_{1}(r,t=0)={p}_{2}(r,t=0)={p}_{3}(r,t=0)={p}_{i}$$

### Dimensionlessness of mathematical models

According to the three-zone radial composite well testing model of the late production well of mixed gas flooding, the dimensionless variables introduced in this section are all based on the physical parameters of the initial crude oil in the crude oil area, and the expressions of the relevant dimensionless parameters are:

There is no factor pressure in each district:

13 $$\:\begin{array}{c}{P}_{nD}=\frac{kh\left({p}_{i}-{p}_{n}\right)}{1.842\times\:1{0}^{-3}q{u}_{o}{B}_{o}}\begin{array}{cc}&\:n=\text{1,2},3\end{array} \end{array}$$

14 $$\:\text{D}\text{i}\text{m}\text{e}\text{n}\text{s}\text{i}\text{o}\text{n}\text{l}\text{e}\text{s}\text{s}\:\text{t}\text{i}\text{m}\text{e}: \, {t}_{D}=\frac{3.6kt}{\varphi\:{C}_{to}{\mu\:}_{o}{r}_{w}^{2}}$$

15 $$\:\text{D}\text{i}\text{m}\text{e}\text{n}\text{s}\text{i}\text{o}\text{n}\text{l}\text{e}\text{s}\text{s}\:\text{r}\text{a}\text{d}\text{i}\text{u}\text{s}: \, \:{r}_{D}=\frac{r}{{r}_{w}}$$

16 $$\:\begin{array}{c}Dimensionless\:wellbore\:reservoir\:coefficient: \, {C}_{D}=\frac{{C}_{w}}{2\pi\:\varphi\:{c}_{to}h{r}_{w}^{2}} \end{array}$$

#### Other relevant parameters

Fluid fluidity ratio at the interface between crude oil fluidity and transition zone in the crude oil zone:

17 $$\:{M}_{12}=\frac{{\left(k/\mu\:\right)}_{1}}{{\left(k/\mu\:\right)}_{2}}, \, \begin{array}{cc}&\:(r={R}_{1D})\end{array}$$

The ratio of crude oil fluidity in the crude oil area to fluid fluidity in the pure gas area:

18 $$\:{M}_{13}=\frac{{\left(k/\mu\:\right)}_{1}}{{\left(k/\mu\:\right)}_{3}}$$

The ratio of fluid storage coefficient at the interface between crude oil fluidity and transition zone in the crude oil zone:

19 $$\:{F}_{12}=\frac{{\left(\phi\:{c}_{t}\right)}_{1}}{{\left(\phi\:{c}_{t}\right)}_{2}}, \, \begin{array}{cc}&\:(r={R}_{1D})\end{array}$$

The ratio of crude oil storage capacity coefficient in crude oil area to pure gas area:

20 $$\:{F}_{13}=\frac{{\left(\phi\:{c}_{t}\right)}_{1}}{{\left(\phi\:{c}_{t}\right)}_{3}}, \, \begin{array}{cc}&\:(r={R}_{2D})\end{array}$$

Fluid conductivity at the interface between the crude oil zone and the transition zone:

21 $$\:{\eta\:}_{12}=\frac{{M}_{12}}{{F}_{12}}, \, \begin{array}{cc}&\:(r={R}_{1D})\end{array}$$

Fluid conductivity coefficient at the interface between the transition zone and the pure gas zone:

22 $$\:{\eta\:}_{13}=\frac{{M}_{13}}{{F}_{13}}, \, \begin{array}{cc}&\:(r={R}_{2D})\end{array}$$

The dimensionless mathematical model can be obtained by bringing the dimensionless variable Eq. (13) ~ (22) into the mathematical model of well testing in the later stage of the mixed gas flooding production well:

#### Seepage differential equations

23 $$\:\text{C}\text{r}\text{u}\text{d}\text{e}\:\text{o}\text{i}\text{l}\:\text{z}\text{o}\text{n}\text{e}: \, \:\frac{{\partial\:}^{2}{P}_{1D}}{\partial\:{r}_{D}^{2}}+\frac{1}{{r}_{D}}\frac{\partial\:{P}_{1D}}{\partial\:{r}_{D}}=\frac{\partial\:{P}_{1D}}{\partial\:{t}_{D}},\begin{array}{cc}&\:1\le\:{r}_{D}<{R}_{1D}\end{array}$$

The nonlinear variation characteristics of mixed oil and gas fluidity and storage capacity coefficient in the transition zone are described by Eqs. (1) and (2), and then the differential equation of nonlinear seepage of mixed oil and gas in the transition zone can be obtained

24 $$\:\begin{array}{c}Transition\:Zone: \, \frac{{\partial\:}^{2}{P}_{2D}}{\partial\:{r}_{D}^{2}}+\frac{1-\theta\:}{{r}_{D}}\frac{\partial\:{P}_{2D}}{\partial\:{r}_{D}}={\eta\:}_{12}{\left(\frac{{r}_{D}}{{R}_{1D}}\right)}^{-\theta\:+I}\frac{\partial\:{P}_{2D}}{\partial\:{t}_{D}},\begin{array}{cc}&\:{R}_{1D}\le\:{r}_{D}<{R}_{2D}\end{array}\end{array}$$

25 $$\:\text{P}\text{u}\text{r}\text{e}\:\text{G}\text{a}\text{s}\:\text{Z}\text{o}\text{n}\text{e}:\:\frac{{\partial\:}^{2}{P}_{3D}}{\partial\:{r}_{D}^{2}}+\frac{1}{{r}_{D}}\frac{\partial\:{P}_{3D}}{\partial\:{r}_{D}}={\eta\:}_{13}\frac{\partial\:{P}_{3D}}{\partial\:{t}_{D}},\begin{array}{cc}&\:{R}_{2D}\le\:{r}_{D}<{R}_{eD}\end{array}$$

#### Inner boundary conditions

26 $$\:({r}_{D}\frac{\partial\:{P}_{1D}}{\partial\:{r}_{D}}{)}_{{r}_{D}=1}=-1$$

#### Outer boundary conditions

27 $$\:\text{I}\text{n}\text{f}\text{i}\text{n}\text{i}\text{t}\text{e}\:\text{s}\text{t}\text{r}\text{a}\text{t}\text{a}: \, {P}_{3D}({r}_{D}\to\:{\infty\:},{t}_{D})=0$$

28 $$\:\begin{array}{c}Circular\:constant\:pressure\:boundary: \, {P}_{3D}\left({r}_{D}={R}_{eD},{t}_{D}\right)=0\end{array}$$

29 $$\:\text{C}\text{i}\text{r}\text{c}\text{u}\text{l}\text{a}\text{r}\:\text{c}\text{l}\text{o}\text{s}\text{e}\text{d}\:\text{b}\text{o}\text{r}\text{d}\text{e}\text{r}\text{s}: \, \frac{\partial\:{P}_{3D}}{\partial\:{r}_{D}}({r}_{D}={R}_{eD},{t}_{D})=0$$

#### Interface conditions

The flow rate equality criterion is followed at the interface between the crude oil zone and the transition zone, and the pressure change characteristics at the interface are described by the “interface skin”:

30 $$\:{\left({P}_{1D}\left({r}_{D}\right)\right)}_{\left({r}_{D}={R}_{1D},{t}_{D}\right)}={P}_{2D}-{\left({S}_{1}{r}_{D}\frac{\partial\:{P}_{2D}}{\partial\:{r}_{D}}\right)}_{\left({r}_{D}={R}_{1D},{t}_{D}\right)}$$

31 $$\:{\left(\frac{\partial\:\left({P}_{2D}\right)}{\partial\:{r}_{D}}\right)}_{({r}_{D}={R}_{1D},{t}_{D})}={\left({M}_{12}{\left(\frac{{r}_{D}}{{R}_{1D}}\right)}^{-\theta\:}\frac{\partial\:\left({P}_{1D}\right)}{\partial\:{r}_{D}}\right)}_{({r}_{D}={R}_{1D},{t}_{D})}$$

At the interface between the transition zone and the pure gas zone, the flow rate equality criterion is followed, and the pressure change characteristics at the interface are described by “interfacial skin”.

32 $$\:{\left({P}_{2D}\left({r}_{D}\right)\right)}_{\left({r}_{D}={R}_{2D},{t}_{D}\right)}={P}_{3D}-{\left({S}_{1}{r}_{D}\frac{\partial\:{P}_{3D}}{\partial\:{r}_{D}}\right)}_{\left({r}_{D}={R}_{2D},{t}_{D}\right)}$$

33 $$\:{\left(\frac{\partial\:\left({P}_{3D}\right)}{\partial\:{r}_{D}}\right)}_{({r}_{D}={R}_{2D},{t}_{D})}={\left(\frac{{M}_{13}}{{M}_{12}}{\left(\frac{{r}_{D}}{{R}_{1D}}\right)}^{\theta\:}\frac{\partial\:\left({P}_{2D}\right)}{\partial\:{r}_{D}}\right)}_{({r}_{D}={R}_{2D},{t}_{D})}$$

#### Initial

The pressure is equal everywhere in the reservoir at the initial moment:

34 $$\:{P}_{1D}({r}_{D},{t}_{D}=0)={P}_{2D}({r}_{D},{t}_{D}=0)={P}_{3D}({r}_{D},{t}_{D}=0)=0$$

### Mathematical model solving

The above dimensionless mathematical model and boundary conditions are transformed by the following formula:

35 $$\:{\overline{P}}_{D}={\int\:}_{0}^{\infty\:}{e}^{-s{t}_{D}}{P}_{D}({r}_{D},{t}_{D})d{t}_{D}$$

Then, we can get the general solution of the Bessel function for the virtual quantities in different regions as: $\:{r}_{D}$

36 $$\:{\overline{P}}_{1D}={A}_{1}{I}_{0}\left({r}_{D}\sqrt{s}\right)+{A}_{2}{K}_{0}\left({r}_{D}\sqrt{s}\right)$$

37 $$\:{\overline{P}}_{2D}={A}_{3}{r}_{D}^{\gamma\:}{I}_{v}\left(\delta\:{r}_{D}^{\beta\:}\right)+{A}_{4}{r}_{D}^{\gamma\:}{K}_{v}\left(\delta\:{r}_{D}^{\beta\:}\right)$$

38 $$\:{\overline{P}}_{3D}={A}_{5}{I}_{0}\left({r}_{D}\sqrt{{\eta\:}_{13}s}\right)+{A}_{6}{K}_{0}\left({r}_{D}\sqrt{{\eta\:}_{13}s}\right)$$

Where:

$$\:\gamma\:=\frac{-\theta\:}{2}\begin{array}{cc}&\:\beta\:=\frac{-\theta\:+I+2}{2}\end{array}\begin{array}{cc}&\:\upsilon\:=\frac{-\theta\:}{-\theta\:+I+2}\end{array}\begin{array}{cc}&\:\delta\:=\end{array}\frac{\sqrt{{R}_{1D}^{-I+\theta\:}{\eta\:}_{12}s}}{\beta\:}$$

Substituting Eq. (36) into the inner boundary condition Eq. (26) yields:

39 $$\:{a}_{11}{A}_{1}+{a}_{12}{A}_{2}+{a}_{13}{A}_{3}+{a}_{14}{A}_{4}+{a}_{15}{A}_{5}+{a}_{16}{A}_{6}=1$$

Where:

40 $$\:{a}_{11}=-s\sqrt{s}{I}_{1}\left(\sqrt{s}\right)$$

41 $$\:{a}_{12}=s\sqrt{s}{K}_{1}\left(\sqrt{s}\right)$$

42 $$\:{a}_{13}=0$$

43 $$\:{a}_{14}=0$$

44 $$\:{a}_{15}=0$$

45 $$\:{a}_{16}=0$$

Substituting Eqs. (36) and (37) into the interface connection condition 30 yields:

46 $$\:{a}_{21}{A}_{1}+{a}_{22}{A}_{2}+{a}_{23}{A}_{3}+{a}_{24}{A}_{4}+{a}_{25}{A}_{5}+{a}_{26}{A}_{6}=0$$

Where:

47 $$\:{a}_{21}={I}_{0}\left({R}_{D1}\sqrt{s}\right)$$

48 $$\:{a}_{22}={K}_{0}\left({R}_{D1}\sqrt{s}\right)$$

49 $$\:{a}_{23}=-{R}_{1D}^{\gamma\:}{I}_{v}\left({R}_{1D}^{\beta\:}\xi\:\right)+{S}_{1}{R}_{1D}\left[\gamma\:{R}_{1D}^{\gamma\:-1}{I}_{v}\left({R}_{1D}^{\beta\:}\xi\:\right)+{R}_{1D}^{\gamma\:}\beta\:{R}_{1D}^{\beta\:-1}\xi\:{I}_{v}^{{\prime\:}}\left({R}_{1D}^{\beta\:}\xi\:\right)\right]$$

50 $$\:{a}_{24}=-{R}_{1D}^{\gamma\:}{K}_{v}\left({R}_{1D}^{\beta\:}\xi\:\right)+{S}_{1}{R}_{1D}\left[\gamma\:{R}_{1D}^{\gamma\:-1}{K}_{v}\left({R}_{1D}^{\beta\:}\xi\:\right)+{R}_{1D}^{\gamma\:}\beta\:{R}_{1D}^{\beta\:-1}\xi\:{K}_{v}^{{\prime\:}}\left({R}_{1D}^{\beta\:}\xi\:\right)\right]$$

51 $$\:{a}_{25}=0$$

52 $$\:{a}_{26}=0$$

Further simplifying Eq. (49) and Eq. (50) respectively yields:

53 $$\:{a}_{23}=-{R}_{1D}^{\gamma\:}{I}_{v}\left({R}_{1D}^{\beta\:}\xi\:\right)+\xi\:\beta\:{S}_{1}{R}_{1D}^{\gamma\:+\beta\:}{I}_{v-1}\left({R}_{1D}^{\beta\:}\xi\:\right)$$

54 $$\:{a}_{24}=-{R}_{1D}^{\gamma\:}{K}_{v}\left({R}_{1D}^{\beta\:}\xi\:\right)-\xi\:\beta\:{S}_{1}{R}_{1D}^{\gamma\:+\beta\:}{K}_{v-1}\left({R}_{1D}^{\beta\:}\xi\:\right)$$

Substituting Eq. (36) and Eq. (37) into the interface condition (30) yields:

55 $$\:{a}_{31}{A}_{1}+{a}_{32}{A}_{2}+{a}_{33}{A}_{3}+{a}_{34}{A}_{4}+{a}_{35}{A}_{5}+{a}_{36}{A}_{6}=0$$

Where:

56 $$\:{a}_{31}=-{M}_{12}\sqrt{s}{I}_{1}\left({R}_{1D}\sqrt{s}\right)$$

57 $$\:{a}_{32}={M}_{12}\sqrt{s}{K}_{1}\left({R}_{1D}\sqrt{s}\right)$$

58 $$\:{a}_{33}=\gamma\:{R}_{1D}^{\gamma\:-1}{I}_{v}\left({R}_{1D}^{\beta\:}\xi\:\right)+\beta\:{R}_{1D}^{\gamma\:+\beta\:-1}\xi\:{I}_{v}^{{\prime\:}}\left({R}_{1D}^{\beta\:}\xi\:\right)$$

59 $$\:{a}_{34}=\gamma\:{R}_{1D}^{\gamma\:-1}{K}_{v}\left({R}_{1D}^{\beta\:}\xi\:\right)+\beta\:{R}_{1D}^{\gamma\:+\beta\:-1}\xi\:{K}_{v}^{{\prime\:}}\left({R}_{1D}^{\beta\:}\xi\:\right)$$

60 $$\:{a}_{35}=0$$

61 $$\:{a}_{36}=0$$

Equation (58) and Eq. (59) above can be simplified to:

62 $$\:{a}_{33}=\xi\:\beta\:{R}_{1D}^{\gamma\:+\beta\:-1}{I}_{v-1}\left({R}_{1D}^{\beta\:}\xi\:\right)$$

63 $$\:{a}_{34}=-\xi\:\beta\:{R}_{1D}^{\gamma\:+\beta\:-1}{K}_{v-1}\left({R}_{1D}^{\beta\:}\xi\:\right)$$

Substituting Eq. (37) and Eq. (38) into the interface condition (31) yields:

64 $$\:{a}_{41}{A}_{1}+{a}_{42}{A}_{2}+{a}_{43}{A}_{3}+{a}_{44}{A}_{4}+{a}_{45}{A}_{5}+{a}_{46}{A}_{6}=0$$

Where:

65 $$\:{a}_{41}=0$$

66 $$\:{a}_{42}=0$$

67 $$\:{a}_{43}={R}_{2D}^{\gamma\:}{I}_{v}\left({R}_{2D}^{\beta\:}\xi\:\right)$$

68 $$\:{a}_{44}={R}_{2D}^{\gamma\:}{K}_{v}\left({R}_{2D}^{\beta\:}\xi\:\right)$$

69 $$\:{a}_{45}=-{I}_{0}\left({R}_{2D}\sqrt{{\eta\:}_{13}s}\right)+{S}_{2}{R}_{2D}\sqrt{{\eta\:}_{13}s}\left){I}_{1}\right({R}_{2D}\sqrt{{\eta\:}_{13}s})$$

70 $$\:{a}_{46}=-{K}_{0}\left({R}_{2D}\sqrt{{\eta\:}_{13}s}\right)-{S}_{2}{R}_{2D}\sqrt{{\eta\:}_{13}s}\left){K}_{1}\right({R}_{2D}\sqrt{{\eta\:}_{13}s})$$

Substituting Eq. (37) and Eq. (38) into the interface condition (32) yields:

71 $$\:{a}_{51}{A}_{1}+{a}_{52}{A}_{2}+{a}_{53}{A}_{3}+{a}_{54}{A}_{4}+{a}_{55}{A}_{5}+{a}_{56}{A}_{6}=0$$

Where:

72 $$\:{a}_{51}=0$$

73 $$\:{a}_{52}=0$$

74 $$\:{a}_{53}=\gamma\:{R}_{2D}^{\gamma\:-1}{I}_{v}\left({R}_{2D}^{\beta\:}\xi\:\right)+\beta\:{R}_{2D}^{\gamma\:+\beta\:-1}\xi\:{I}_{v}^{{\prime\:}}\left({R}_{2D}^{\beta\:}\xi\:\right)$$

75 $$\:{a}_{54}=\gamma\:{R}_{2D}^{\gamma\:-1}{K}_{v}\left({R}_{2D}^{\beta\:}\xi\:\right)+\beta\:{R}_{2D}^{\gamma\:+\beta\:-1}\xi\:{K}_{v}^{{\prime\:}}\left({R}_{2D}^{\beta\:}\xi\:\right)$$

76 $$\:{a}_{55}=-\frac{{M}_{12}}{{M}_{13}}\left(\frac{{R}_{2D}}{{R}_{1D}}{)}^{-\theta\:}\sqrt{{\eta\:}_{13}s}{I}_{1}\right({R}_{2D}\sqrt{{\eta\:}_{13}s})$$

77 $$\:{a}_{56}=\frac{{M}_{12}}{{M}_{13}}\left(\frac{{R}_{2D}}{{R}_{1D}}{)}^{-\theta\:}\sqrt{{\eta\:}_{13}s}{K}_{1}\right({R}_{2D}\sqrt{{\eta\:}_{13}s})$$

Equation (74) and Eq. (75) above can be simplified to:

78 $$\:{a}_{53}=\xi\:\beta\:{R}_{2D}^{\gamma\:+\beta\:-1}{I}_{v-1}\left({R}_{2D}^{\beta\:}\xi\:\right)$$

79 $$\:{a}_{54}=-\xi\:\beta\:{R}_{2D}^{\gamma\:+\beta\:-1}{K}_{v-1}\left({R}_{2D}^{\beta\:}\xi\:\right)$$

Substituting Eq. (38) into the outer boundary conditions (27), (28), and (29) respectively yields:

80 $$\:{a}_{61}{A}_{1}+{a}_{62}{A}_{2}+{a}_{63}{A}_{3}+{a}_{64}{A}_{4}+{a}_{65}{A}_{5}+{a}_{66}{A}_{6}=0$$

Where:

81 $$\:{a}_{61}=0$$

82 $$\:{a}_{62}=0$$

83 $$\:{a}_{63}=0$$

84 $$\:{a}_{64}=0$$

When the boundary is infinite:

85 $$\:{a}_{45}={a}_{55}={a}_{65}={a}_{66}=0$$

When the outer boundary is a constant pressure boundary:

86 $$\:{a}_{65}={I}_{0}\left({R}_{eD}\sqrt{{\eta\:}_{13}s}\right)$$

87 $$\:{a}_{66}={K}_{0}\left({R}_{eD}\sqrt{{\eta\:}_{13}s}\right)$$

When the outer boundary is closed:

88 $$\:{a}_{65}={I}_{1}\left({R}_{eD}\sqrt{{\eta\:}_{13}s}\right)$$

89 $$\:{a}_{66}=-{K}_{1}\left({R}_{eD}\sqrt{{\eta\:}_{13}s}\right)$$

The coefficients to be determined can be obtained by constructing the coefficient matrix of Eqs. (39), (46), (55), (64), (71), and (80): $\:{A}_{1}\:{A}_{2}\:{A}_{3}\:{A}_{4}\:{A}_{5}\:{A}_{6}$

90 $$\:{A}_{1}=\frac{{H}_{1}}{H}$$

91 $$\:{A}_{2}=\frac{{H}_{2}}{H}$$

92 $$\:{A}_{3}=\frac{{H}_{3}}{H}$$

93 $$\:{A}_{4}=\frac{{H}_{4}}{H}$$

94 $$\:{A}_{5}=\frac{{H}_{5}}{H}$$

95 $$\:{A}_{6}=\frac{{H}_{6}}{H}$$

式中:

$$\:H=\left|\begin{array}{cccccc}{a}_{11}&\:{a}_{12}&\:0&\:0&\:0&\:0\\\:{a}_{21}&\:{a}_{22}&\:{a}_{23}&\:{a}_{24}&\:0&\:0\\\:{a}_{31}&\:{a}_{32}&\:{a}_{33}&\:{a}_{34}&\:0&\:0\\\:0&\:0&\:{a}_{43}&\:{a}_{44}&\:{a}_{45}&\:{a}_{46}\\\:0&\:0&\:{a}_{53}&\:{a}_{54}&\:{a}_{55}&\:{a}_{56}\\\:0&\:0&\:0&\:0&\:{a}_{65}&\:{a}_{66}\end{array}\right|\:,\:{H}_{1}=\left|\begin{array}{cccccc}1&\:{a}_{12}&\:0&\:0&\:0&\:0\\\:0&\:{a}_{22}&\:{a}_{23}&\:{a}_{24}&\:0&\:0\\\:0&\:{a}_{32}&\:{a}_{33}&\:{a}_{34}&\:0&\:0\\\:0&\:0&\:{a}_{43}&\:{a}_{44}&\:{a}_{45}&\:{a}_{46}\\\:0&\:0&\:{a}_{53}&\:{a}_{54}&\:{a}_{55}&\:{a}_{56}\\\:0&\:0&\:0&\:0&\:{a}_{65}&\:{a}_{66}\end{array}\right|\:,$$

$$\:{H}_{2}=\left|\begin{array}{cccccc}{a}_{11}&\:1&\:0&\:0&\:0&\:0\\\:{a}_{21}&\:0&\:{a}_{23}&\:{a}_{24}&\:0&\:0\\\:{a}_{31}&\:0&\:{a}_{33}&\:{a}_{34}&\:0&\:0\\\:0&\:0&\:{a}_{43}&\:{a}_{44}&\:{a}_{45}&\:{a}_{46}\\\:0&\:0&\:{a}_{53}&\:{a}_{54}&\:{a}_{55}&\:{a}_{56}\\\:0&\:0&\:0&\:0&\:{a}_{65}&\:{a}_{66}\end{array}\right|\:,\:{H}_{3}=\left|\begin{array}{cccccc}{a}_{11}&\:{a}_{12}&\:1&\:0&\:0&\:0\\\:{a}_{21}&\:{a}_{22}&\:0&\:{a}_{24}&\:0&\:0\\\:{a}_{31}&\:{a}_{32}&\:0&\:{a}_{34}&\:0&\:0\\\:0&\:0&\:0&\:{a}_{44}&\:{a}_{45}&\:{a}_{46}\\\:0&\:0&\:0&\:{a}_{54}&\:{a}_{55}&\:{a}_{56}\\\:0&\:0&\:0&\:0&\:{a}_{65}&\:{a}_{66}\end{array}\right|\:,$$

$$\:{H}_{4}=\left|\begin{array}{cccccc}{a}_{11}&\:{a}_{12}&\:0&\:1&\:0&\:0\\\:{a}_{21}&\:{a}_{22}&\:{a}_{23}&\:0&\:0&\:0\\\:{a}_{31}&\:{a}_{32}&\:{a}_{33}&\:0&\:0&\:0\\\:0&\:0&\:{a}_{43}&\:0&\:{a}_{45}&\:{a}_{46}\\\:0&\:0&\:{a}_{53}&\:0&\:{a}_{55}&\:{a}_{56}\\\:0&\:0&\:0&\:0&\:{a}_{65}&\:{a}_{66}\end{array}\right|\:,\:{H}_{5}=\left|\begin{array}{cccccc}{a}_{11}&\:{a}_{12}&\:0&\:0&\:1&\:0\\\:{a}_{21}&\:{a}_{22}&\:{a}_{23}&\:{a}_{24}&\:0&\:0\\\:{a}_{31}&\:{a}_{32}&\:{a}_{33}&\:{a}_{34}&\:0&\:0\\\:0&\:0&\:{a}_{43}&\:{a}_{44}&\:0&\:{a}_{46}\\\:0&\:0&\:{a}_{53}&\:{a}_{54}&\:0&\:{a}_{56}\\\:0&\:0&\:0&\:0&\:0&\:{a}_{66}\end{array}\right|\:,$$

$$\:{H}_{6}=\left|\begin{array}{cccccc}{a}_{11}&\:{a}_{12}&\:0&\:0&\:0&\:1\\\:{a}_{21}&\:{a}_{22}&\:{a}_{23}&\:{a}_{24}&\:0&\:0\\\:{a}_{31}&\:{a}_{32}&\:{a}_{33}&\:{a}_{34}&\:0&\:0\\\:0&\:0&\:{a}_{43}&\:{a}_{44}&\:{a}_{45}&\:0\\\:0&\:0&\:{a}_{53}&\:{a}_{54}&\:{a}_{55}&\:0\\\:0&\:0&\:0&\:0&\:{a}_{65}&\:0\end{array}\right|$$,

By substituting the coefficients into Eqs. (36), (37) and (38), the pressure calculation formula in the Laplace space at any position in the crude oil zone, the transition zone, the mixed oil and gas, and the pure gas zone can be obtained. At the same time, the formula for calculating the bottom hole pressure can be obtained by substituting Eq. (36) into the inner boundary condition Eq. (26): $\:{A}_{1}\:{A}_{2}\:{A}_{3}\:{A}_{4}\:{A}_{5}\:{A}_{6}$.

96 $$\:{\left({\overline{P}}_{1D}\right)}_{{r}_{D}=1}={A}_{1}{I}_{0}\left(\sqrt{s}\right)+{A}_{2}{K}_{0}\left(\sqrt{s}\right)$$

The solution of the downhole pressure under the Lapl ace space under the influence of the wellbore reservoir coefficient CD and the skin effect S can be obtained by Eq. (97)$\:{\overline{P}}_{WD}$^23^

97 $$\:{\overline{P}}_{WD}=\frac{s{\overline{P}}_{1D}\left(\sqrt{s}\right)+S}{s\left\{1+{C}_{D}s\left[s{\overline{P}}_{1D}\left(\sqrt{s}\right)+S\right]\right\}}$$

## Analysis of pressure dynamics

**Fig. 3:**
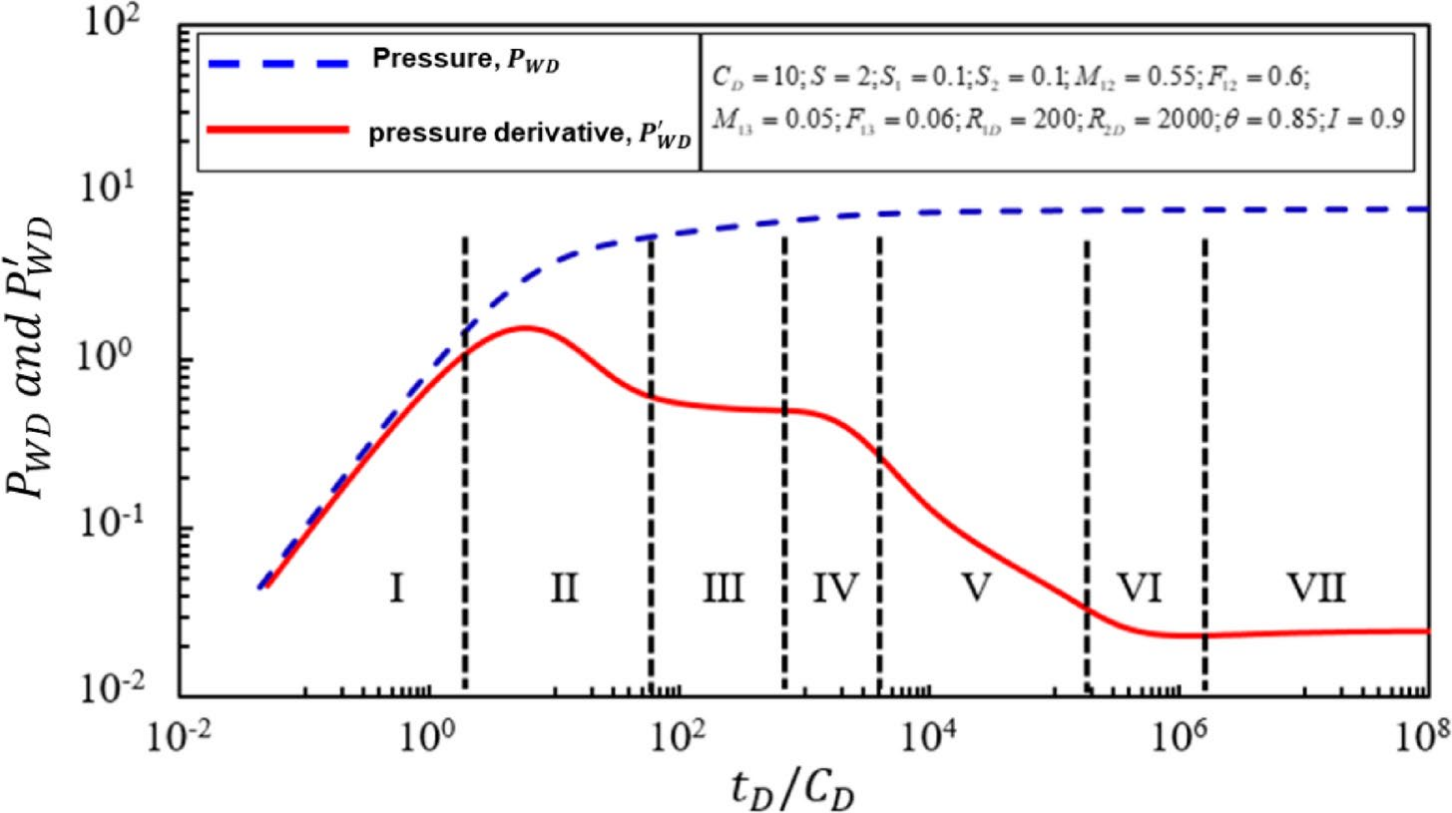
Log-log typical curve for late production well with triple-zone composite fluid.

To analyze the flow characteristics of mixed gas flooding production wells in the later stage, the Stehfest numerical inversion technique is further used to invert the solution in bottom hole pressure Eq. 97 under certain parameters (Fig. 3)^24^, and the dimensionless double-logarithmic bottom hole pressure dynamic response characteristic curves of mixed gas flooding injection downhole pressure in the late downhole period are obtained. Analyzing the research results in Fig. 3, it can be seen that the seepage stages of the typical curve of pressure and pressure derivative are divided into$\:\:{P}_{WD}\sim{t}_{D}/{C}_{D}{P}_{WD}^{{\prime\:}}({t}_{D}/{C}_{D})\sim{t}_{D}/{C}_{D}$ 7 seepage stages.

- In the first stage, in the wellbore reservoir control seepage stage, the pressure derivative curve and pressure curve are straight lines with a slope of 1, and the two straight lines are coincident straight lines through the coordinate origin.
- In the second stage, the seepage characteristic segment is mainly controlled by the epidermal coefficient, which is characterized by the upper “convexity”. In addition, the seepage stage is also affected by the wellbore reservoir coefficient.
- In the third stage, the radial seepage stage of the crude oil area, the pressure derivative curve is a horizontal straight line of 0.5 because the fluidity and storage capacity coefficient of the crude oil in the reservoir in the crude oil area are constant values. In addition, this horizontal segment can only appear if the crude oil zone is large enough, and if the width of the crude oil zone is small, the pressure derivative curve usually appears as a downward “concave” characteristic.
- In the fourth stage is the transitional seepage stage between the oil zone and the transitional zone. This stage is mainly controlled by three parameters: the skin factor $\:{S}_{1}$ at the interface, the flow capacity ratio $\:{M}_{12}$ between the oil zone and the transitional zone, and the comprehensive storage capacity coefficient ratio $\:{F}_{12}$ at the interface between the oil zone and the transitional zone. The pressure derivative curve shows a downward trend.
- In the fifth stage, in the concave flow stage of mixed oil and gas in the transitional zone. Due to the semi-radial variation characteristics of flow capacity and storage coefficient with a power-law, the pressure derivative curve exhibits a deviation from 0.5 $\:{M}_{12}$ toward a downward-sloping straight line. The straight-line slope is closely related to the power-law variation indices of the storage capacity/flow capacity $\:\left(\theta\:,I\right)$.
- In the sixth stage, the transitional seepage stage between the transitional zone and the pure gas zone. This stage is mainly controlled by three parameters: the skin factor $\:{S}_{2}$ at the interface between the transitional zone and the pure gas zone, the flow capacity ratio $\:{M}_{13}$ between the oil zone and the pure gas zone, and the comprehensive storage capacity coefficient ratio $\:{F}_{13}$ at the interface between the oil zone and the pure gas zone. The pressure derivative curve shows a slightly “convex”.
- In the seventh stage, the radial flow stage in the pure gas zone system. The pressure derivative curve is equal to 0.5 $\:{M}_{13}$ and appears as a horizontal straight line.

## Parameter sensitivity analysis

### Radius of the crude oil zone

**Fig. 4:**
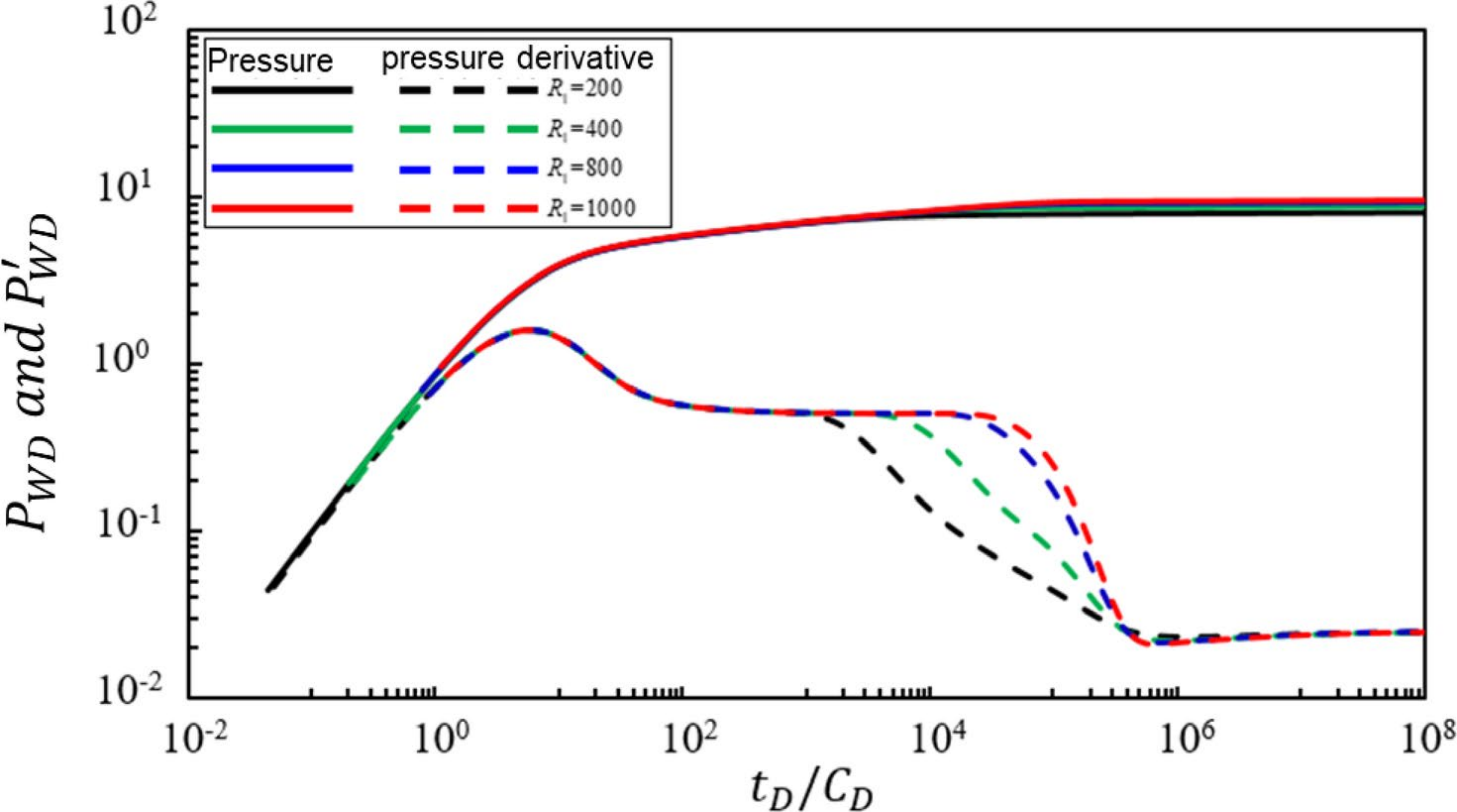
Effect of the dimensionless radius of the oil zone on bottom-hole pressure dynamics.

Figure 4 illustrates the impact of the dimensionless radius $\:{R}_{1}$ of the oil zone’s flow boundary on the bottomhole pressure dynamics of a production well. Under the condition that other parameters remain constant, the pressure and pressure derivative characteristic curves were calculated for $\:{R}_{1}$ values of 200, 400, 800, and 1000. Analysis of Fig. 4 indicates the following conclusions: The dimensionless radius $\:{R}_{1}$ of the oil zone primarily affects the third, fourth, and fifth seepage stages. As $\:{R}_{1}$ increases: (1) The duration of radial flow in the oil zone becomes longer. (2) The corresponding duration of concave radial flow in the mixed oil-gas zone of the transitional zone becomes shorter. (3) While the change in the radius of the oil zone leads to variations in flow capacity and the distribution characteristics of the storage coefficient, the declining trend remains unchanged. Specifically, as the dimensionless radius $\:{R}_{1}$ of the oil zone increases, the downward slope of the straight-line characteristic segment in the concave radial flow of the mixed oil-gas zone in the transitional zone does not exhibit a significant change.

### Radius of the transition zone$\:{R}_{2}$

**Fig. 5:**
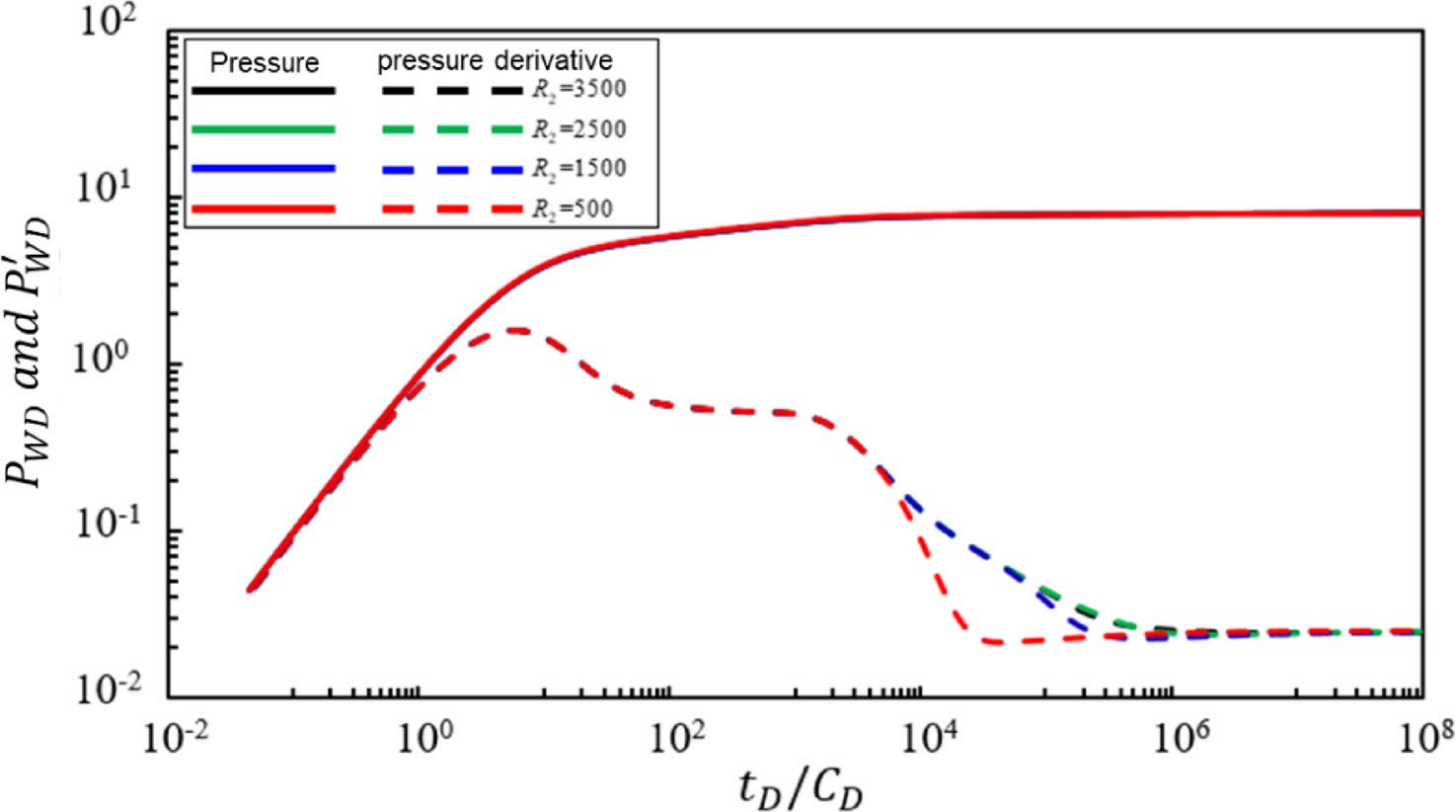
Effect of the dimensionless boundary radius of the transition region on bottom-hole pressure dynamics.

Figure 5 shows the impact of the dimensionless radius $\:{R}_{2}$ of the mixed oil-gas zone in the transitional region on bottomhole pressure dynamics. Under the condition that other parameters remain constant, the pressure and pressure derivative characteristic curves were calculated for $\:{R}_{2}$ values of 3500, 2500, 1500, and 500. Analysis of Fig. 5 reveals the following conclusions: The dimensionless radius $\:{R}_{2}$ of the mixed oil-gas zone in the transitional region primarily affects the fifth, sixth, and seventh seepage stages. As $\:{R}_{2}$ increases: (1) The duration of radial flow in the mixed oil-gas zone in the transitional region becomes longer. (2) The duration of radial flow in the pure gas zone becomes shorter. Additionally, when the dimensionless radius $\:{R}_{2}$ of the mixed oil-gas zone in the transitional region equals 500, the characteristic segment of radial flow in the mixed oil-gas zone in the transitional region disappears, and a sharp downward trend is observed.

### Power law change index of mixed oil and gas fluidity in the transition zone

**Fig. 6:**
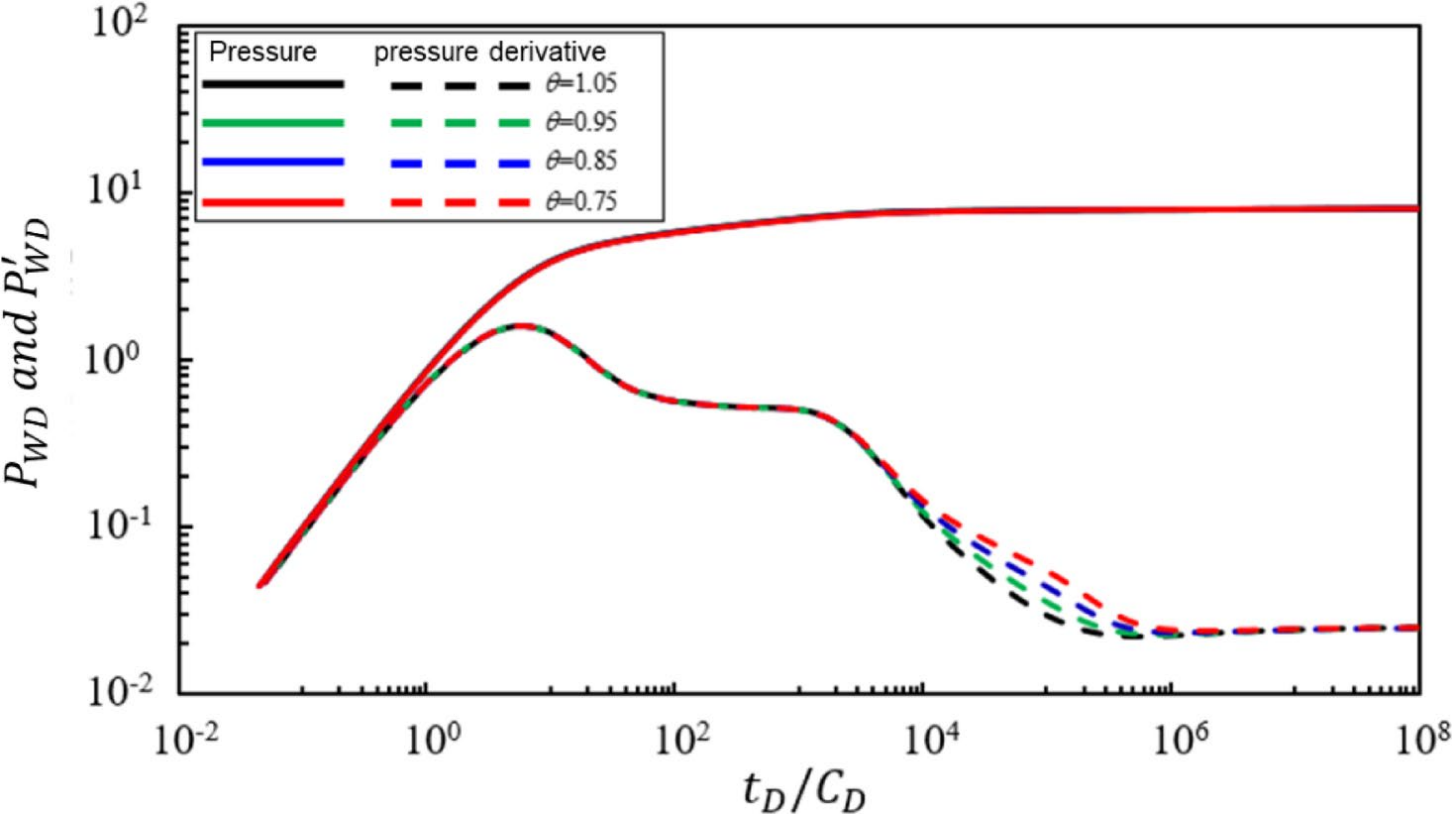
Effect of the power-law exponent of mobility in the transition region $\:\left(\theta\:\right)$ on bottom-hole pressure dynamics

Figure 6 shows the impact of the power-law variation index $\:\theta\:$ of the fluid mobility for the mixed oil-gas zone in the transitional region on the bottomhole pressure dynamics of a production well. Under the condition that other parameters remain constant, the pressure and pressure derivative characteristic curves were calculated for $\:\theta\:$ values of 1.05, 0.95, 0.85, and 0.75. Analysis of Fig. 6 reveals the following conclusions: The power-law variation index $\:\theta\:$ of fluid mobility in the mixed oil-gas zone primarily affects the fifth seepage stage. As $\:\theta\:$ increases: (1) The slope of the straight-line characteristic segment of radial flow in the mixed oil-gas zone in the transitional region becomes steeper. (2) The duration of radial flow in the mixed oil-gas zone in the transitional region does not show a significant change.

### Power law variation index of mixed oil and gas storage capacity coefficient in the transition zone

**Fig. 7:**
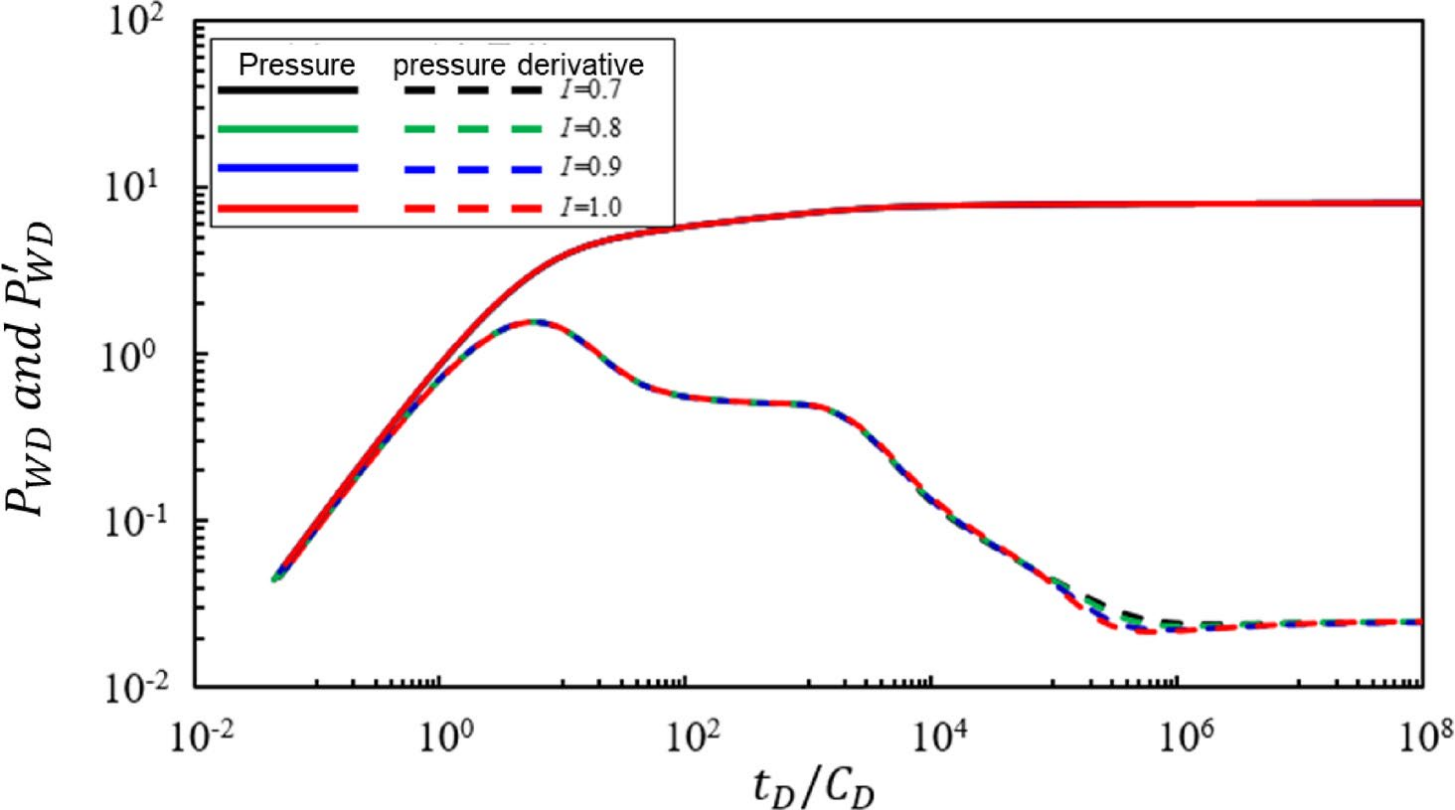
Effect of the power-law exponent of storativity coefficient in the transition region (I) on bottom-hole pressure dynamics.

Figure 7 shows the impact of the power-law variation index I of the storage capacity coefficient for the mixed oil-gas zone in the transitional region on bottomhole pressure dynamics. Under the condition that other parameters remain constant, the pressure and pressure derivative characteristic curves were calculated for I values of 1, 0.9, 0.8, and 0.7. Analysis of Fig. 7 reveals the following conclusions: The power-law variation index III of the storage capacity coefficient primarily affects the fifth and sixth seepage stages. As I increases: (1) The slope of the straight-line characteristic segment of radial flow in the mixed oil-gas zone in the transitional region becomes steeper. (2) The duration of radial flow in the mixed oil-gas zone in the transitional region does not show significant change. Compared with the power-law variation index $\:\theta\:$ of mobility, the power-law variation index I has a relatively weaker effect on the slope of the straight-line segment of radial flow in the mixed oil-gas zone in the transitional region.

## Conclusion

1. To address the complex flow characteristics of miscible gas-flooded production wells in later stages, this study innovatively develops a three-zone radial composite well-testing model. By dividing the formation into an oil zone, transition zone, and pure gas zone, the model pioneers the application of power-law functions to describe nonlinear spatiotemporal variations in fluid mobility and storativity within the transition zone. This advancement overcomes traditional composite models’ limitations in homogeneity assumptions and linear dependencies. The introduction of interfacial skin coefficients further enhances the model’s capability to characterize pressure jump effects at zone interfaces, significantly improving its representation of dynamic behaviors in heterogeneous reservoirs.
2. Through Laplace transform and Stehfest numerical inversion methods, analytical solutions for the nonlinear flow model are achieved, revealing seven-stage flow characteristics in pressure transient curves of miscible gas-flooded wells. These stages include oil-zone radial flow, transition-zone power-law concave-slope flow, and pure gas-zone horizontal stabilization. Compared with conventional well-testing models, this approach provides more precise characterization of complex multiphase fluid interactions and dynamic evolution during miscible gas flooding processes.
3. Parameter sensitivity analysis elucidates key mechanisms: Oil-zone radius (R₁) governs radial flow duration, transition-zone radius (R₂) determines gas-oil percolation scope, while power-law exponents (θ and I) regulate slope variation and morphological differences in transition-zone pressure derivative curves. These findings establish theoretical foundations for dynamic monitoring and nonlinear parameter inversion in gas-flooded wells.
4. This research expands the applicability of well-test analysis in heterogeneous, nonlinear flow scenarios, providing crucial technical support for evaluating gas flooding efficiency, optimizing injection-production strategies, and enhancing oil recovery. Future studies could integrate multi-physics coupling mechanisms with field-measured data to advance quantitative characterization of transition-zone dynamics.
5. There are still some limitations in this study: although the influence of a single parameter is discussed in this paper, the actual field scene often involves the combination of parameters. For example, increasing the size of the reservoir area while reducing the mobility nonlinearity may prolong the duration of the radial flow in the reservoir area, but reduce the concave slope of the transition zone. Future research should explore the interaction between such parameters, so as to provide guidance for parameter inversion under complex conditions.

## Data Availability

The datasets generated during and/or analysed during the current study are available from the corresponding author on reasonable request.

## List of symbols

${\left(k/\mu \right)}_{1}$: Fluid fluidity in the crude oil zone in the late stage of gas flooding in production wells $\left(mD\cdot c{p}^{-1}\right)$
${\left(k/\mu \right)}_{2}$: Fluid fluidity in the transition zone in the late stage of gas flooding in production wells $\left(mD\cdot c{p}^{-1}\right)$
${\left(k/\mu \right)}_{3}$: Fluid flow in gas region $\left(mD\cdot c{p}^{-1}\right)$
${\left(\varnothing {t}_{t}\right)}_{1}$: Crude oil storage capacity coefficient in the late stage of gas flooding of production wells $(MP{a}^{-1})$
${\left(\varnothing {t}_{t}\right)}_{2}$: Fluid storage capacity coefficient in the transition zone in the later stage of gas flooding in production wells $(MP{a}^{-1})$
${\left(\varnothing {t}_{t}\right)}_{3}$: The energy storage coefficient of gas zone in the later stage of gas flooding in production wells $(MP{a}^{-1})$
$\theta$: Power law variation index of fluid flow in the transition zone in the late stage of gas flooding in production wells (dimensionless)
$I$: Power law variation index of storage capacity coefficient in the transition zone in the late stage of gas flooding in production wells (dimensionless)
${M}_{12}$: Interface fluidity ratio between crude oil zone and transition zone in the later stage of gas flooding in production wells (dimensionless)
${M}_{13}$: The mobility ratio of crude oil area to pure gas area in the later stage of gas flooding in production wells (dimensionless)
${F}_{12}$: Storage-capacity ratio at the interface between the crude oil zone and the transition zone in the late stage of gas flooding in production wells (dimensionless)
${F}_{13}$: The ratio of energy storage coefficient between crude oil area and pure gas area in the later stage of gas flooding in production wells (dimensionless)
${\eta }_{12}$: Interface fluid conductivity coefficient of crude oil zone and transition zone (dimensionless)
${\eta }_{13}$: Interface fluid conductivity coefficient between transition zone and pure gas zone (dimensionless)
${S}_{1},{S}_{2}$: Interface skin factor (dimensionless)
${C}_{D}$: Wellbore storage coefficient (dimensionless)
${t}_{D}$: Dimensionless time (dimensionless)
${P}_{nD}$: The dimensionless pressure in zone n ( n = 1,2,3n = 1,2,3 correspond to crude oil zone, transition zone and pure gas zone, respectively) (dimensionless)
$\overline{{P }_{D}}$: Dimensionless pressure in Laplace space (dimensionless)
${r}_{D}$: Dimensionless distance from the reservoir to the injection well (dimensionless)
${R}_{1}$: The dimensionless radius of fluid flow in crude oil region (dimensionless)
${R}_{2}$: The dimensionless radius of mixed oil and gas in the transition zone (dimensionless)
${R}_{e}$: Supply radius (dimensionless)
${R}_{1D}$: Dimensionless radius of crude oil area (dimensionless)
${R}_{2D}$: Dimensionless radius of gas-oil transition zone (dimensionless)
${R}_{D1}$: Inner zone dimensionless radius (dimensionless)
${R}_{D2}$: Outer zone dimensionless radius (dimensionless)
${R}_{De}$: Dimensionless supply radius (dimensionless)

## References

[1] Li, P. & Hu, M. A study on the effect of new surfactant proportions on the recovery improvement of carbonate reservoir. *Appl. Sci.* **14**, 4028 (2024).

[2] Tobing, E. Ml. Changing wellbore storage in gas well testing. *Sci. Contrib. Oil Gas*. **31**, 40–48 (2022).

[3] Miller, C. C., Dyes, A. B. & Hutchinson, C. A. The Estimation of permeability and reservoir pressure from bottom hole pressure build-up characteristics. *J. Pet. Technol.* **2**, 91–104 (1950).

[4] Gobran, B. D., Brigham, W. E. & Ramey, H. J. Absolute permeability as a function of confining pressure, pore pressure, and temperature. *SPE Form. Eval*. **2**, 77–84 (1987).

[5] Gringarten, A. C., Ramey, H. J. & Raghavan, R. Applied pressure analysis for fractured wells. *J. Pet. Technol.* **27**, 887–892 (1975).

[6] Bourdet, D., Ayoub, J. A. & Plrard, Y. M. Use of pressure derivative in well-test interpretation. *SPE Form. Eval*. **4**, 293–302 (1989).

[7] Fevang, Ø. & Whitson, C. H. Modeling gas-condensate well deliverability. *SPE Reserv. Eng.* **11**, 221–230 (1996).

[8] Xu, S. & Lee, W. J. Two-phase well test analysis of gas condensate reservoirs. In *SPE Annual Technical Conference and Exhibition* SPE-56483-MSSPE (1999). 10.2118/56483-MS

[9] Bozorgzadeh, M. & Gringarten, A. C. New estimate for the radius of a condensate bank from well test data using dry gas pseudo-pressure. In *SPE Annual Technical Conference and Exhibition* SPE-89904-MS (2004). 10.2118/89904-MS

[10] Kgogo, T. C. & Gringarten, A. C. Comparative well-test behaviours in low-permeability lean, medium-rich, and rich gas-condensate reservoirs. In *SPE Annual Technical Conference and Exhibition* SPE-134452-MSSPE (2010). 10.2118/134452-MS

[11] Yin, F., Cheng, S., Bai, W., Wang, Y. & Liu, X. Single-well simulation for horizontal wells in fractured gas condensate reservoirs. *ACS Omega*. 10.1021/acsomega.3c10360 (2024). 10.1021/acsomega.3c10360 PMC11024962 38645378

[12] Liang, X., Lin, Y. & Fan, C. The characteristic of water injection response to complex reservoir. In *Proceedings of the International Conference on Advances in Energy, Environment and Chemical Science* (Atlantis Press, 2016). 10.2991/aeecs-16.2016.7

[13] LaFitte, C. & James, L. A. Assessment of oil recovery methods for reservoirs in the flemish pass basin. in *Day 2 Thu, March 17*, D021S025R002 (SPE, 2022). 10.2118/208906-MS

[14] Wang, X. et al. Study on interaction characteristics of injected natural gas and crude oil in a high saturation pressure and low-permeability reservoir. *Processes* **11**, 2152 (2023).

[15] Nguyen, T. V. & Tran, X. V. Gas-assisted gravity drainage process for improved oil recovery in Bao Den fractured basement reservoir. *Sci. Technol. Dev. J.* **19**, 161–168 (2016).

[16] Mohammadpour, M., Behnoud, P. & Khorsand Movaghar, M. R. Develop an empirical flow rate correlation to model wellbore storage phenomenon for wells produced at a constant Wellhead pressure. *Sci. Rep.* **13**, 17726 (2023). 37853044 10.1038/s41598-023-44678-3 PMC10584919

[17] Ahammad, J. M., Rahman, M. A., Butt, S. D. & Alam, J. M. Integrated wellbore-reservoir modeling based on 3D navier–stokes equations with a coupled CFD solver. *J. Pet. Explor. Prod. Technol.* **14**, 2539–2554 (2024).

[18] Garipova, A. et al. Numerical simulation study of huff-n-puff hydrocarbon gas injection parameters for enhanced shale oil recovery. *Energies* **16**, 1555 (2023).

[19] Luo, J. & Wang, L. Research on gas channeling identification method for gas injection development in high-pressure heterogeneous reservoir. *Processes* **10**, 2366 (2022).

[20] Youssef, A. A. & Matthäi, S. K. Upscaled dynamic relative permeability for unstable CO2 flow in stratified porous media. *Transp. Porous Media*. **143**, 657–680 (2022).

[21] Feng, Y. et al. Influence of geomechanics parameters on stress sensitivity in fractured reservoir. *Front. Earth Sci.* **11**, 1134260 (2023).

[22] Peng, X., Liu, Y., Liang, B. & Du, Z. Interface condition for the Darcy velocity at the water-oil flood front in the porous medium. *PLOS One*. **12**, e0177187 (2017). 28542612 10.1371/journal.pone.0177187 PMC5441608

[23] Li, X., Liu, S., Chen, Q., Su, Y. & Sheng, G. Evaluation of volume fracturing effect of horizontal wells in tight oil reservoirs considering complex fracture networks. *PETROLEUM Drill. TECHNIQUES*. **47**, 73–82 (2019).

[24] Brzeziński, D. W. Review of numerical methods for NumILPT with computational accuracy assessment for fractional calculus. *Appl. Math. Nonlinear Sci.* **3**, 487–502 (2018).

